# Comprehensive Transcriptome Analysis of *GS3* Near-Isogenic Lines During Panicle Development in Rice (*Oryza sativa* L.)

**DOI:** 10.3389/fgene.2022.857143

**Published:** 2022-03-01

**Authors:** Wenhua Liang, Fengqin Hu, Weicong Qi, Chunfang Zhao, Tao Chen, Cailin Wang, Yuanda Lv, Yadong Zhang

**Affiliations:** ^1^ Institute of Food Crops, Jiangsu Academy of Agricultural Sciences, Nanjing, China; ^2^ Jiangsu High Quality Rice R&D Center, Nanjing Branch of China National Center for Rice Improvement, Nanjing, China; ^3^ Excellence and Innovation Center, Jiangsu Academy of Agricultural Sciences, Nanjing, China; ^4^ Key Laboratory of Jiangsu Province for Agrobiology, Nanjing, China

**Keywords:** rice, GS3, NIL, panicle, g-protein, hormones

## Abstract

Panicle architecture is an important agronomic trait in rice that affects rice yields and quality. The *GRAIN SIZE 3* (*GS3*) locus has been identified as a major quantitative trait locus (QTL) affecting grain length and weight. The current understanding of the function of the *GS3* gene, especially concerning the regulatory mechanism of panicle development, is still in its infancy. In this study, we generated *GS3* near-isogenic lines (*NILs*) by successive crossing and backcrossing of TD70 (large grain) with Kasalath (small grain), using Kasalath as the recurrent parent. To identify potential transcription dynamic changes in rice panicle formation and grain shape, we deeply analyzed transcriptional profiles for the *NILs* (*NIL*-*GS3* and *NIL*-*gs3*) at three different panicle developmental stages (S, M, and L). A total of 887, 1,768, and 1,478 differentially expressed genes (DEGs) were identified at stages S, M, and L, respectively. We also found 542 differential expressed long non-coding RNAs (lncRNAs). Co-expression analysis further revealed significant clusters associated with different development periods in *NIL*-*gs3* lines. Gene Ontology and KEGG enrichment analysis revealed G-protein signaling and hormones pathway were successively activated at the M and L stages of *NIL-gs3*, which indicated activation of the G-protein signaling pathway might trigger the down-streaming hormone signaling transduction. we found that other hormones such ABA, Auxin, CK were significantly enriched in the L stage in the *NIL-gs3*. We highlighted the synergistic interplay of G-protein and multiple hormones signaling pathways and their essential roles in regulating rice panicle formation and the grain shape. Our study provides an invaluable resource for further molecular mechanistic studies that affect rice grain size and provide new insight for directed selection by marker-assisted backcross breeding.

## Introduction

Rice is one of the most important food crops globally, feeding more than half of the world’s population. Grain weight is determined by grain size and grain filling degree, whereas the yield is based on grain weight, panicle quantity, and quantity per panicle. It is important to improve grain size in rice breeding, as it is closely related to yield, and quality. Thus, investigating the gene expression regulation associated with grain development can potentially provide an avenue for improving crop yield and quality.

Many genetic studies examining grain-shape traits have been carried out in the past few decades, and many grain shape-related genes and quantitative trait loci (QTL) have been cloned (Huang et al., 2013; Wang Y et al., 2019). Among them, genes associated with grain length have been identified, including *GS3(Grain Size 3), qGL3(Grain Length 3), GL7/GW7(Grain Length/Width 7), GL4(Grain Length 4), TGW3*/*GL3.3(Grain Length 3.3), GS9(Grain Size 9)*, and *GLA(Grain Length and Awn)* (Mao et al., 2010; Zhang et al., 2012; Wang S et al., 2015; Wang Y et al., 2015; Wu et al., 2017; Xia et al., 2018; Ying et al., 2018; Zhao et al., 2018; Zhang et al., 2019). The *GS3* gene is located in the pericentromeric region of rice chromosome 3 and encodes a predicted membrane protein that regulates grain size by controlling cell proliferation. Furthermore, this QTL has been shown to control grain size and play a role as a negative regulator concerning grain and organ size (Fan et al., 2006; Mao et al., 2010).

During rice grain development, multiple grain shape-related genes, and their interactions affect the developmental outcome. Another gene, *GW8*, which belongs to the SBP transcription factor family, has been shown to promote cell proliferation and grain filling when overexpressed and contributes to grain widening and increased yields. Furthermore, *GW8* can bind to the *GL7/GW7/SLG* promoter to inhibit its transcription and regulate cell proliferation in spikelet glumes (Wang et al., 2012; Wang S et al., 2015). Additionally, both *GLW7* and *GW8* positively regulate the cell size of the grain hull to increase the rice grain length and yield. Small and round seed 5 (SRS5) encodes an α-tubulin, with a one amino acid change (p.Arg308Leu) that reduces the rice seed length by hindering cell elongation. Furthermore, although *GLW7* can interact with *SRS5* to regulate grain size, the underlying mechanism remains unclear. However, *GLW7* has been shown to bind the *DEP1* promoter, and significantly affect panicle length and primary branch number. Additionally, the *GLW7* and *GS3* genes have been shown to act independently (Si et al., 2016). Thus, a complete characterization of these genes can aid in improved molecular breeding.

*GW2* and *GW5* positively regulate the expression of *GS3*, and *GW5* can be down-regulated by repressing *GW2* transcription. Moreover, *GS3* can alleviate the effect of *GW5* on grain length, and *GW5* also can alleviate the effect of *GS3* on grain width (Yan et al., 2011). Furthermore, *GS3* and *qGL3* are negative regulators of grain length and weight, with a *GS3*/*qGL3* loss-of-function leading to a long grain length. In near-isogenic lines (*NILs*), *GS3,* and *qGL3* have exhibited a cumulative effect on rice grain length regulation, possibly because of a common receptor protein kinase signal pathway (Gao et al., 2015).

In plants, heterotrimeric G-proteins are conserved signaling molecules that play essential roles in multiple signaling pathways. The 3 G protein subunits (α, β, and γ) have different functions in rice. An α subunit (*RGA1/D1*) loss-of-function leads to a dwarf phenotype and reduced grain size and lowers BR sensitivity, while a decreased β subunit (*RGB1*) expression also results in a short-grain size and dwarf phenotype. The γ subunit comprises five homologous genes (*RGG1, RGG2, qPE9-1/DEP1, GGC2*, and *GS3*) with varying functions. *RGG2* is a negative regulator of plant growth and organ size and interacts with *RGB1* in the gibberellin (GA) pathway. Furthermore, *DEP1* and *GGC2* interact with the β subunit to positively regulate grain length. Although *GS3* itself does not affect grain length, it can inhibit the effects of *DEP1* and *GGC2* by competitively binding the β subunit. Therefore, a *GS3* deletion results in a long-grain phenotype. Additionally, *RGG1* enhances salt tolerance in rice by improving reactive oxygen species (ROS) detoxification (Sun et al., 2018; Miao et al., 2019; Wang Y et al., 2019; Xu et al., 2019). *OsMADS1* acts as a key downstream effector of the βγ G protein dimer, with the γ subunits (*GS3*, *DEP1*) directly interacting with *MADS1* to increase its transcriptional activity thereby affecting downstream target genes and affecting grain morphology. Moreover, the pyramiding of these alleles, *OsMADS1* ^*lgy3*^, *dep1-1*, and *gs3*, can improve rice yield and quality (Liu et al., 2018). In the Nipponbare genome, *GL3.3* (*GSK5*) encodes a GSK3/SHAGGY-like kinase and negatively regulates grain length. Furthermore, *GL3.3* is constitutively expressed in different tissues and organs and exhibits epistatic interactions with *GS3* (Xia et al., 2018).

RNA sequencing (RNA-Seq) enables genome-wide transcriptomic analysis and has been applied to examine rice panicle development, fertility, heterosis, and salinity stress (Zhang et al., 2014; Ke et al., 2016; Guo et al., 2017; Zhang et al., 2018; Wang J et al., 2019). In eukaryotes, long non-coding RNAs (lncRNAs) are produced by RNA polymerases, are greater than 200 bp in length, and have no protein potential (Han et al., 2019). LncRNAs have been implicated in plant growth, reproduction, disease resistance, and stress (Lee et al., 2013; Zhang and Chen, 2013; Lv et al., 2019).

In this study, we used *NILs* to examine dynamic transcriptional changes at different stages with/without *GS3* in rice panicle development using time-course RNA-Seq. We also examined the potential transcriptional regulatory effects of directed selection of the *GS3* gene by marker-assisted backcross breeding (MABB).

## Results

### Variations in *gs3* and Grain Shape Phenotype in *NILs*

**FIGURE 1:**
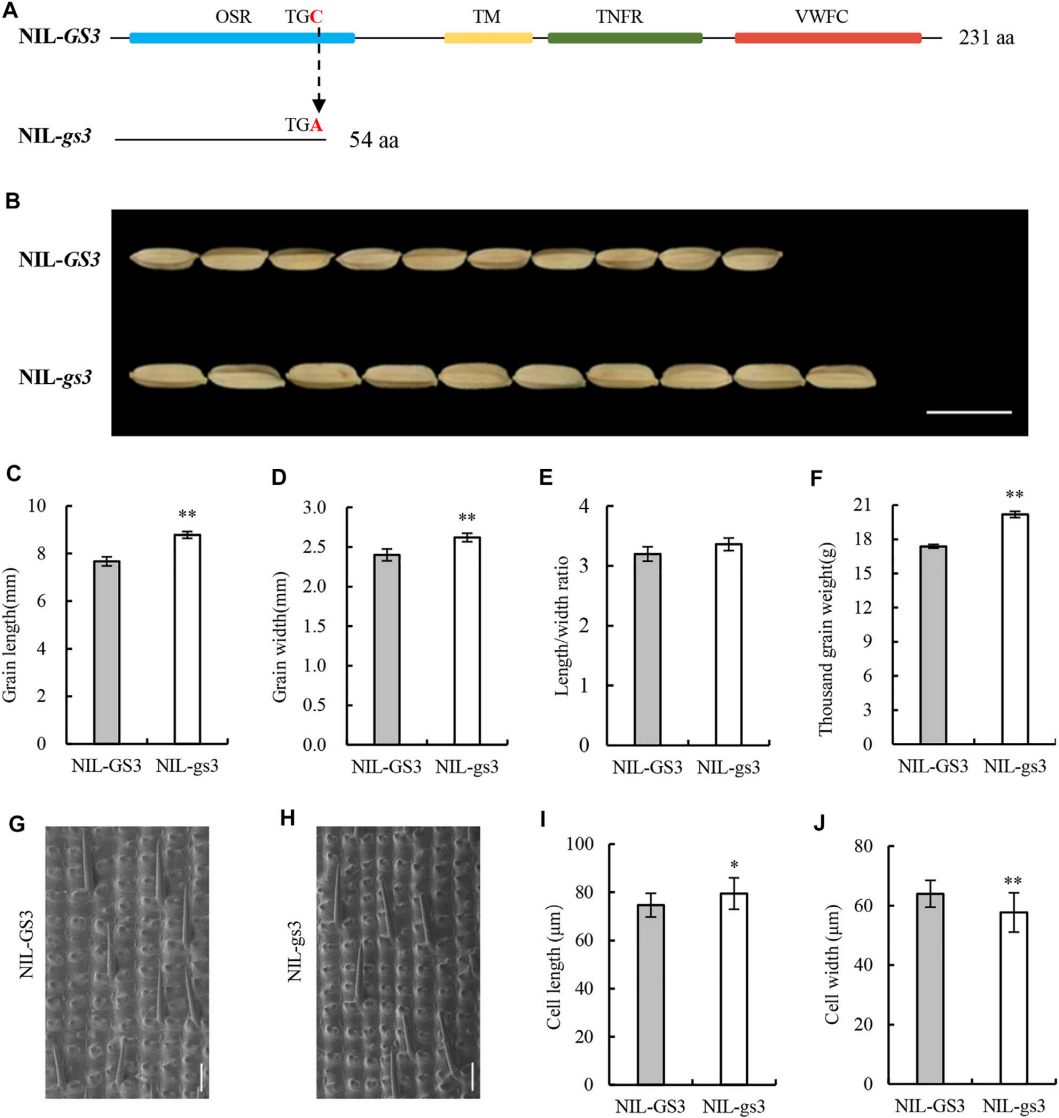
Differences in the *GS3* gene structure and the grain phenotype in *NIL-GS3* and *NIL-gs3.* **(A)** Structural differences in *GS3* between the *NILs*. **(B)** Appearance of mature grains. Bar = 10 mm. Grain **(C)** length, **(D)** width, **(E)** length/width ratio, and **(F)** thousand-grain weight. **(G, H)** Imaging of epidermal cells in the middle glume of mature grains using a scanning electron microscope. Bar = 100 μm. **(I, J)** Cell length and width statistics. Data are displayed as a mean ± SD (*n* = 20). Asterisks indicate significance (Student’s *t*-test, *p < 0.05, **p < 0.01).

Previous studies have confirmed that *GS3* is the major QTL that controls grain length and weight in rice, with an SNP (C→A) mutation in the second exon leading to early termination of translation and a subsequent long-grain phenotype (Fan et al., 2006; Fan et al., 2009; Mao et al., 2010). In *NIL*-*GS3*, the ORF length of *GS3* was 696 bp and encoded 231 amino acids. The domain prediction identified four functional domains: the N-terminal organ size regulator (OSR) domain, a transmembrane domain (TM), a C-terminal TNFR/NGFR family cysteine-rich structure, and a VWFC structure (Figure 1A). In *NIL*-*gs3*, however, the *GS3* locus mutated from C to A at 165 bp, resulting in a premature termination codon (TGC→TGA) and a sequence with only 54 amino acids (Figure 1A). The *GS3* loci in *NIL*-*GS3* and *NIL*-*gs3* were the same as those reported in Zhenshan 97 and Minghui 63, respectively (Mao et al., 2010).

When comparing the grain length, length-to-width ratio, and thousand-grain weight, *NIL*-*gs3* increased by 14.32, 4.69, and 16.17%, respectively, relative to *NIL*-*GS3* (*p* < 0.05). Additionally, the grain width of *NIL*-*gs3* increased by 9.17%, but we did not note any significant difference in grain thickness. Therefore, the *gs3* locus contained in *NIL*-*gs3* had a positive effect on rice grain size (Figures 1B–F; Supplementary Table S1).

To examine the cytological basis of grain size differences in the *GS3* isogenic lines, we examined the morphology of the cells in the middle portion of the glumes of mature *NIL*-*GS3* and *NIL*-*gs3* seeds and compared them using SEM. The results showed that the glume cell length was significantly longer in *NIL*-*gs3* than *NIL*-*GS3* (6.50%, in the grain-length orientation), whereas the cell width decreased significantly (9.77%). Subsequently, the glume epidermal cells in *NIL*-*gs3* appeared to be more slender, and the length-to-width ratio increased. These findings indicated that the *GS3* gene regulates rice grain shape by regulating glume cell morphology (Figure 1G–J; Supplementary Table S1).

### Transcriptome Assembly and Potential Coding Prediction

To comprehensively analyze the transcriptome changes in rice grain development following a *GS3* mutation, we examined three representative stages (S:small panicle size, M:medium panicle size, and L:large panicle size) from the beginning of panicle to the beginning of the heading in the *NILs.* We obtained a total of 114.78 G base pairs, with an average of 6.38 G per sample. We then successfully aligned the reads to the reference genome IRGSP-1.0 using Hisat2. The results showed that the percentage of unique alignments to the genome was between 89.49 and 91.96%, with an average of 90.84% (Supplementary Table S2).

**FIGURE 2:**
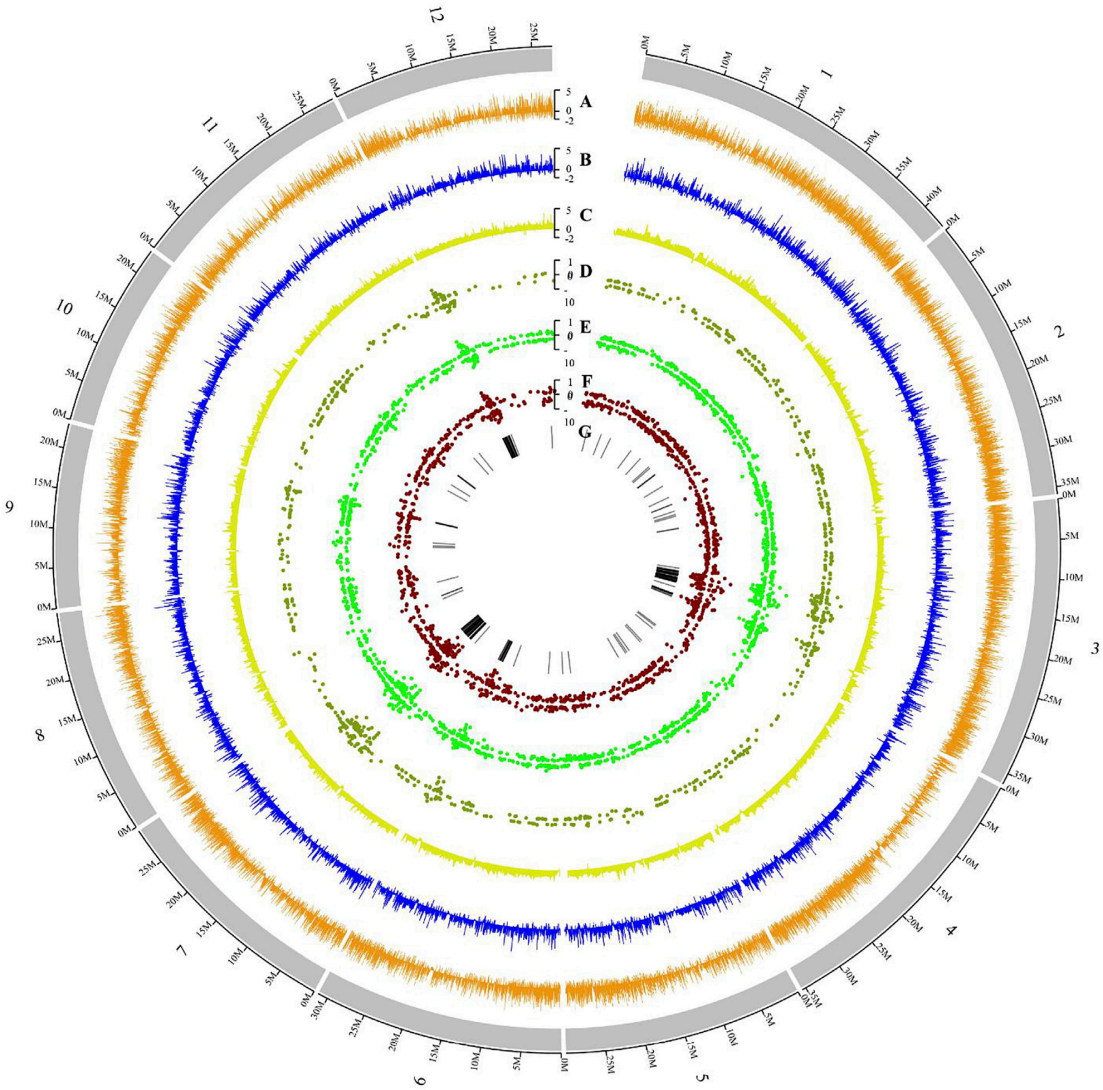
Gene expression distributions in *NIL*-*GS3* and *NIL*-*gs3* during panicle development. **(A)** Distribution of all gene expression profile; **(B)** LncRNA expression profiles; **(C)** LncRNA expression profile. The *Y*-axis shows log10-scaled average FPKM values. Distributions of deferentially expressed genes in *NIL*-*GS3* and *NIL*-*gs3* at the **(D)** S stage **(E)** M stage, and **(F)** L stage. The *Y*-axis shows the Log2 (Fold change). **(G)** Distribution of common DEGs within the three stages.

After RABT assembly, we obtained 85,411 transcripts from 44,668 genes, with 5,936 new transcripts identified in the intergenic regions (Supplementary Table S3; Figure 2). We then predicted coding potentials using CPC2 and PLEK and identified 18,343 transcripts (15,136 genes) as lncRNA candidates. Among them, 4,143 were located in the intergenic regions and 155 were located in introns. These two types of lncRNAs can be collectively called lincRNAs. Additionally, 1,255 lncRNA transcripts overlapped with gene exons, presumably because of alternative splicing (Supplementary Table S3). Quantitative analysis of the expression profiles showed that lncRNA expression, especially lincRNA expression, was lower than that of coding genes, consistent with previous studies (Lv et al., 2019).

### Identification of DEGs Between *NILs*

During rice growth and development, dynamic changes in gene expression occur in different tissues and organs during different developmental stages. At the booting stage, many genes associated with cell proliferation, cell expansion, transcription factors, and hormone regulation modulate inflorescence and panicle morphogenesis (Ke et al., 2016; Wang Y et al., 2019; Zha et al., 2019). When identifying DEGs at the different developmental stages in *NIL*-*GS3*, we identified 5,974 DEGs at the S stage relative to the M stage, 2,395 at the L stage relative to M, and 9,400 at the L stage relative to the S. Among these three groups of DEGs, a total of 742 genes were commonly significantly differently expressed. We also observed this phenomenon in the three stages of spikelet development in *NIL*-*gs3* (Supplementary Table S4). These findings clearly showed the presence of gene expression stratification at different developmental stages.

**FIGURE 3:**
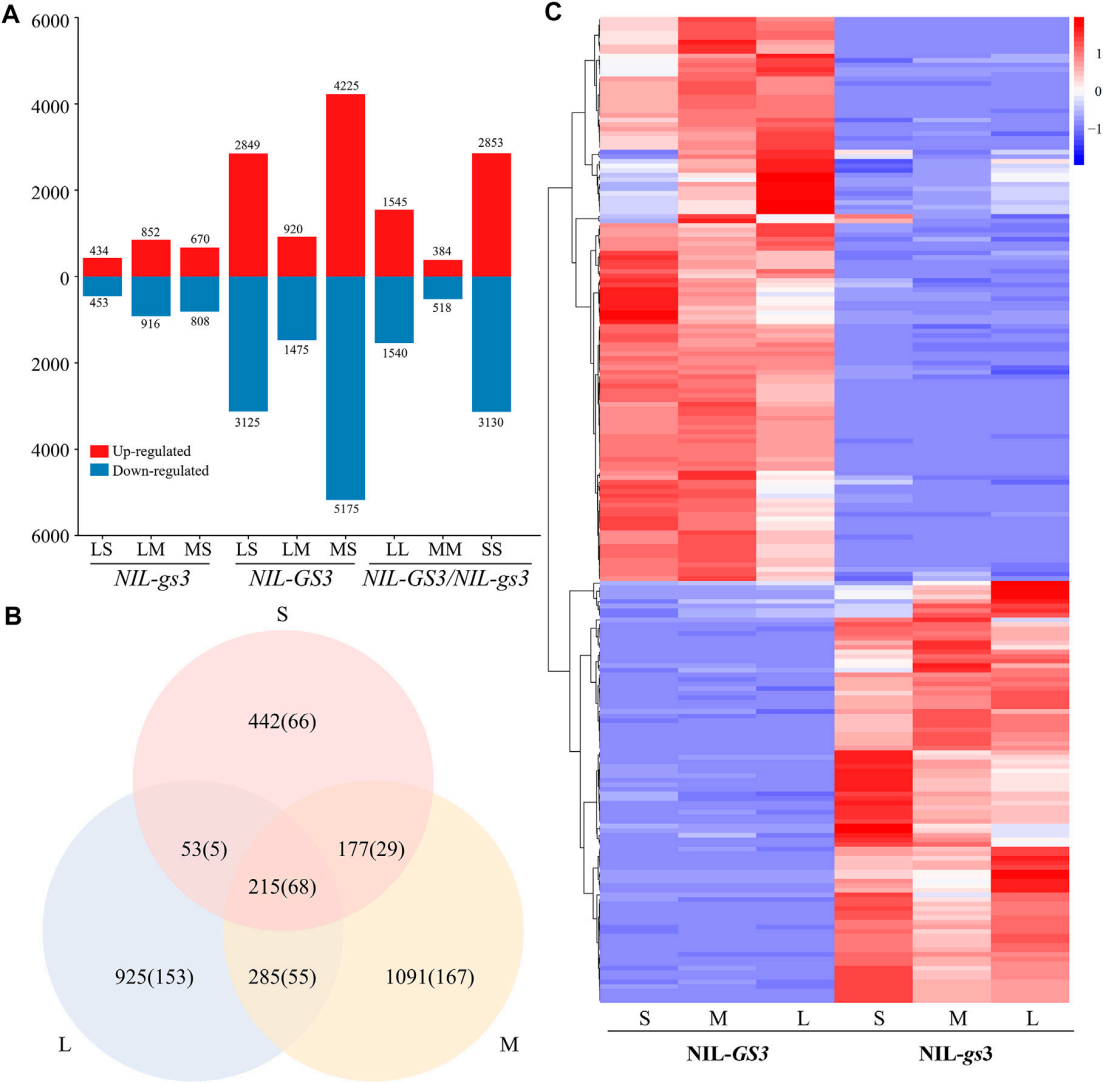
*NIL*-*GS3* and *NIL*-*gs3* differential expression at different stages of panicle development **(A)** Identification of DEGs at different developmental stages. **(B)** Number of differentially expressed lncRNAs at different stages. **(C)** Comparative analysis of DEGs at different developmental stages. The number of lncRNAs is in parentheses. **(D)** Expression profiles of the DEGs for all three stages. FPKM values were normalized to the Z-scores.

To further examine expressional changes during different developmental stages, we compared differential expression between the *NIL* groups at each stage (Figure 3; Supplementary Table S4). During the S stage, we identified 887 DEGs (167 lncRNAs), 434 upregulated (85 lncRNAs), and 453 downregulated (82 lncRNAs) in *NIL*-*gs3* relative to the *NIL*-*GS3*. For the M stage, we identified 1,768 DEGs (318 lncRNAs), 852 upregulated (129 lncRNAs) and 916 downregulated (189 lncRNAs); For the L stage, we identified 1,478 DEGs (280 lncRNAs), 670 upregulated (151 lncRNAs), and 808 down-regulated (129 lncRNAs). Furthermore, the number of DEGs increased with the growth of the panicle, thus suggesting that *GS3* affected the expression of more genes in the mid-late stages of panicle development (Figure 3A). When comparing the young *NIL* panicles at the same stage, 215 genes (66 lncRNAs) were commonly differentially expressed across the three stages. Furthermore, hierarchical clustering analysis also showed a similar expression pattern between the groups (Figures 2, 3C). The number of unique differential genes in the S, M, and L stages were 442 (90 LncRNAs), 1,091 (126 LncRNAs), and 925 (120 LncRNAs), respectively (Figure 3B). To confirm the RNA-Seq findings, we randomly selected ten representatives of differentially expressed transcripts, including two lncRNAs, and eight coding genes. The qRT-PCR results showed that three of the transcripts were significantly upregulated, and five were significantly down-regulated among S, M, and L periods. (Supplementary Figure S1). All changes were consistent with expected results from the RNA-Seq data, thus indicating a high degree of reliability.

### A Dynamic Expression Landscape During Kernel Development

**FIGURE 4:**
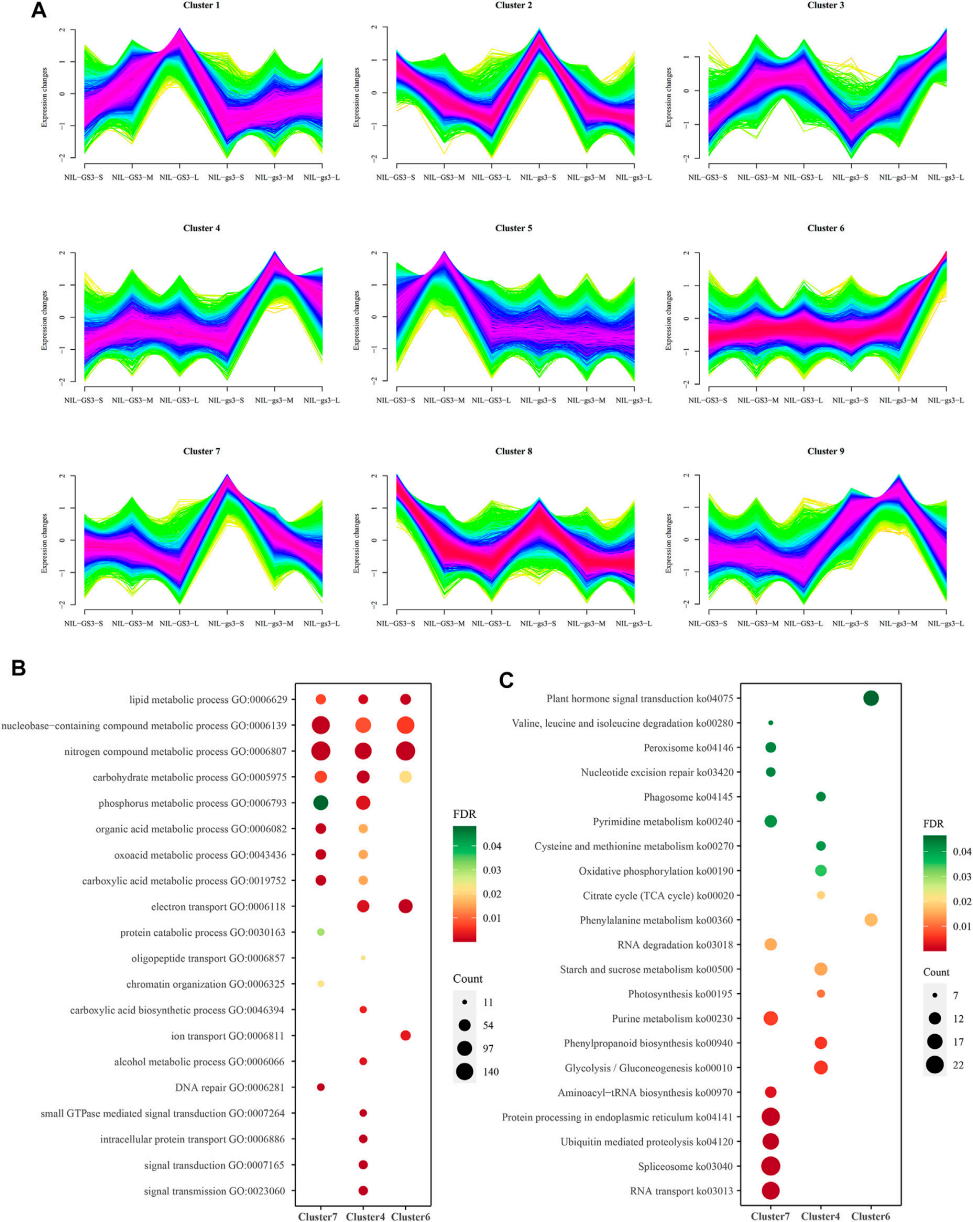
Clustering of expression profiles using a fuzzy clustering algorithm. **(A)** The expression patterns of 9 clusters generated by Mfuzz software. The expression values were represented by normalized Z-score. **(B)** GO enrichment analysis of cluster 7, 4, and 6, corresponding to different stages of *NIL*-*GS3* and *NIL*-*gs3* panicle development. Developmental stages include S (1–3 cm), M (4–6 cm), and L (8–10 cm); **(C)** KEGG enrichment analysis of cluster 7, 4, and 6.

The c-means fuzzy clustering algorithm was employed for clustering gene expression profiles in all developmental stages (Kumar and Futschik, 2007). The fuzzy clustering algorithm could assign a membership value for each gene expression profile of each cluster using different fuzziness values. As a result, nine distinct clusters of temporal patterns representing *NILs* and development stages were generated (Figure 4A; Supplementary Table S5). Among them, cluster 7, cluster 4, and cluster 6 were identified, which represented different development stages (S, M, and L) and significantly upregulated in the corresponding stage of *NIL-gs3*, whereas cluster 8, cluster 5, and cluster 1 are upregulated and showed stage-specific in three stages of *NIL-GS3*.

**FIGURE 5:**
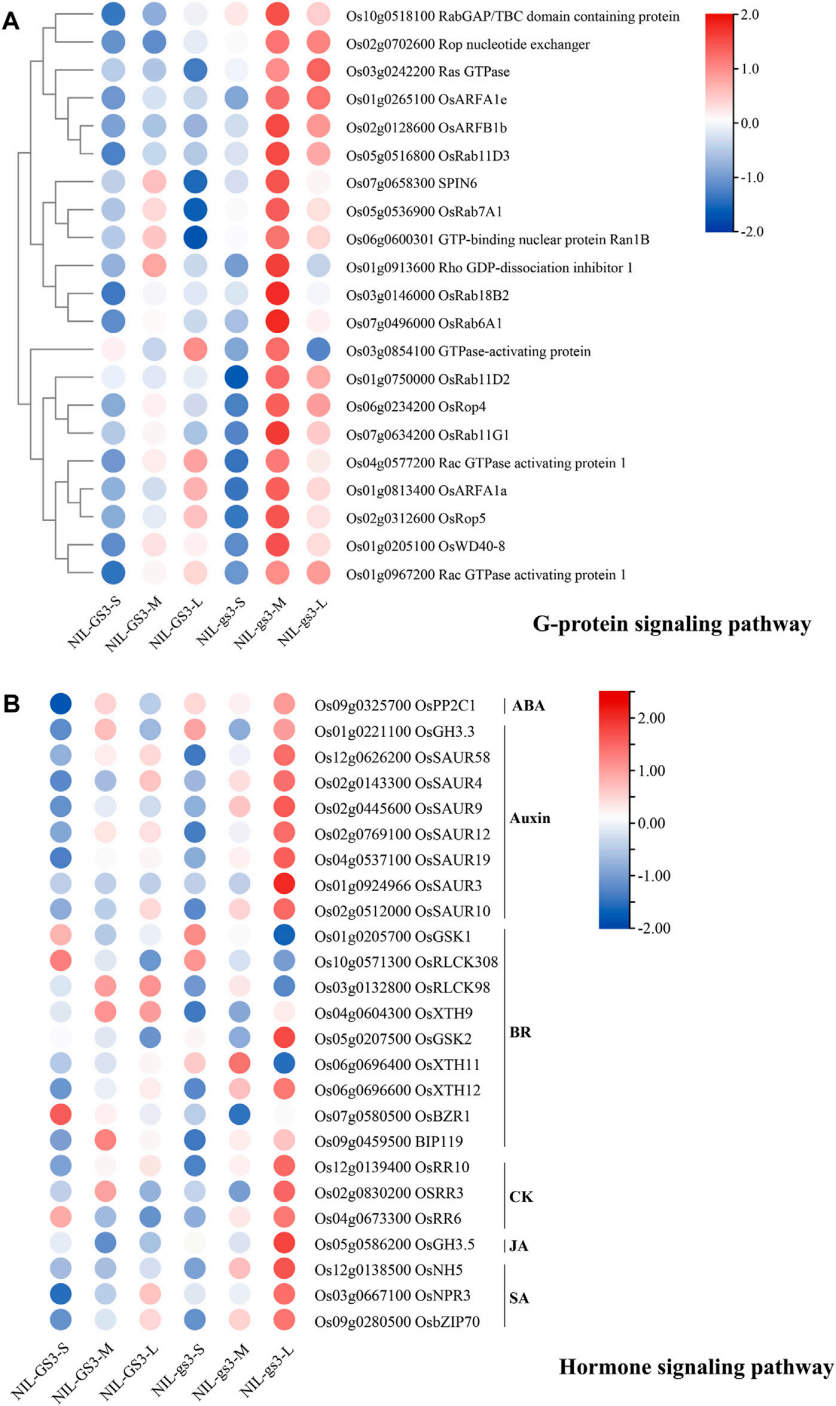
The expression profiles of genes associated with G-protein and hormone signaling pathways. **(A)** The heatmap of genes from the G-protein signaling pathway; **(B)** The heatmap of genes from the G-protein signaling pathway. FPKM values were normalized to the Z-scores.

GO and KEGG enrichment analysis of these 9 clusters showed many enriched biological processes and pathways corresponding to the relevant stages (Figure 4B, Supplementary Table S6). For example, genes associated with nitrogen compound metabolic process, lipid metabolic process, carbohydrate metabolic process, and nucleobase-containing compound metabolic process are highly enriched in cluster 7, 4, and 6, corresponding to three stages (S, M, and L). Those genes associated with the organic acid metabolic process, phosphorus metabolic process, and oxoacid metabolic process were significantly enriched in cluster 7 (S stage) and cluster 4 (M stage). Three gamma subunits, *GS3* (Os03g0407400), *GGC2* (Os08g0456600), and *DEP1* (Os09g0441900), were predominantly expressed at the early stage of panicle development and then rapidly declined (Supplementary Table S3). Interestingly, small GTPase mediated signal transduction, intracellular protein transport, and signal transmission was significantly enriched in Cluster4 (M stage). Meanwhile, starch and sucrose metabolism, Glycolysis, and phenylpropanoid biosynthesis were also enriched explicitly in the M stage. Most genes such as *OsRab6A1, OsRop4, OsWD40,* and *OsRho* associated with the G-protein signaling pathway were significantly upregulated in the M stage (Figure 5A). Besides, genes associated with plant hormone signal transduction such as *OsGSK1, OsRLCK98, OsXTH9, OsGH3.3, and OsRR10* were specifically enriched and upregulated in the L stage (Figure 5B). These results suggested that G-protein and hormone signaling pathways may show a synergistic interplay in regulating panicle development in rice.

## Discussion

In rice, *GS3* is a major QTL that affects grain length and weight. In large-grain varieties, an SNP (C→A) mutation in the second exon of the *GS3* gene leads to premature termination of translation, thus suggesting that *GS3* may function as a negative regulator affecting grain length and weight (Fan et al., 2006; Fan et al., 2009). The phenotypic differences between *NIL*-*GS3* and *NIL*-*gs3* are mainly due to functional differences in the *GS3* alleles because *NIL*-*GS3* produces a functional protein and *NIL*-*gs3* produces a truncated protein and generates a long grain. The structure of the *GS3* locus in the isogenic lines was consistent with the previously reported Zhenshan 97 and Minghui 63 lines (Mao et al., 2010). The *NIL*-*gs*3 group showed an increase in grain length and thousand-grain weight and a slight increase in grain width, with no significant change in grain thickness compared with *NIL*-*GS3* (Figures 1A–E; Supplementary Table S1). These findings further confirmed that the *GS3* locus is closely related to grain length and weight and that this locus has a negative effect on grain size (Mao et al., 2010). When examining the cytological effect of *GS3* in the epidermal cells in glumes, the glume cell length increased, whereas the width decreased in *NIL*-*gs3*; thus, resulting in a more elongated morphology. In addition to affecting the morphology, the *NIL*-*gs3* group also showed an increase in proliferation. Thus, the *gs3* allele carried by *NIL*-*gs3* increased the rice yield and provided an improved grain shape (Figure 1; Supplementary Table S1).

Previous studies that utilized *NILs* also identified many DEGs associated with rice growth and development (Moumeni et al., 2015; Tariq et al., 2018) and panicle development, with many of these DEGs associated with morphological development (Ke et al., 2016; Meng et al., 2017; Wang J et al., 2019). In this study, the observed phenotypic difference between the *NIL*-*GS3* and *NIL*-*gs3* groups predominantly resulted from functional differences. Thus, we examined the panicles of these *NILs* at three different stages (S, M, and L) and constructed transcriptional profiles. Comparative analysis identified DEGs in the *NIL*-*gs3* group at the S (*n* = 887), M (*n* = 1768), and L (*n* = 1,478), thus suggesting that *GS3* had a more significant influence on the middle and late stages of panicle development (Figure 3A). Besides, many differential expressed lncRNAs were also identified and showed dynamic changes during panicle development, which suggested that lncRNAs may also play a regulatory role in rice panicle development.

To better characterize the potential effect of *NIL*-*gs3* in regulating panicle development, we further employed the c-means fuzzy clustering method for partitioning gene clusters that have a similar expression profile (Figure 4A). The clustering information could help identify genes underlining major biological processes and understanding how these genes are involved in complex panicle development. Nine clusters were generated, and clusters 7, 4, and 6 were correlated explicitly with different panicle development stages. Through GO and KEGG enrichment, many biological processes/pathways were significantly enriched across different development stages. G-protein and hormone signal pathways were noteworthy successively activated at the M and L stages of *NIL-gs3* (Figures 4B,C). As shown in Figure 5, genes G-protein signaling pathway were significantly upregulated in the M stage of *NIL*-*gs3,* which indicated that the *GS3* gene, as negative regulators, and activated the G-protein signaling pathway in *NIL*-*gs3* lines (Figure 5A). Genes associated with hormone signal pathways, including Auxin, BR, CK were also significantly upregulated in the L stage of *NIL*-*gs3* (Figure 5B). These results suggested that G-protein coupled with hormone signaling pathway regulated the panicle development and then affected kernel size, consistent with previous reports (Mao et al., 2010; Sun et al., 2018). Previous studies have shown that the BR signaling pathway is highly correlated with cell elongation and growth (Uozu et al., 2000; Jan et al., 2004; Hernandez-Nistal et al., 2010). Herein, *OsGSK1, OsRLCK308* were mainly upregulated in early development. *OsXTH11* was upregulated in the S stage and reached the peak in the M stage, whereas OsXTH12 was upregulated in the M stage and reached the peak in the L stage. Thus, different genes from the BR signaling pathway might play a distinct role in different development stages. Compared with the BR pathway, Auxin, CK, JA, and SA pathways were mainly activated in the L stage in *NIL*-*gs3* lines. These findings suggested that the synergistic interplay of G-protein and hormone signaling pathway might play an essential role in rice panicles development and impact grain size.

## Conclusion

*GS3* is the major QTL that controls grain size in rice. In this study, we identified 887, 1,768, and 1,478 DEGs, including many lncRNAs among three periods when comparing the panicles of *NIL*-*GS3* and *NIL*-*gs3* via RNA-Seq. By further c-means fuzzy clustering, 9 clusters representing distinct expression patterns were generated. Many biological processes or pathways were significantly enriched in different development stages of *NIL*-*gs3* lines. G-protein signaling pathway was mainly activated in the M stage, whereas multiple hormones singling pathways including Auxin, BR, CK, JA, and SA were activated in the L stage. These findings suggested that the difference in grain size between the *NILs* most likely be attributed to the complex synergistic interplay of G-protein and multiple hormone signaling pathways.

## Materials and Methods

### Plant Material Sampling and Grain Shape Trait Measurements

We collected a large grain *Japonica* variety (TD70) and a small grain *Indica* variety (Kasalath) (Zhang et al., 2015). We then constructed *NILs* (*NIL*-*gs3* and *NIL*-*GS3*) using TD70 as a donor and Kasalath as the background by conventional six-generation backcrossing. The *NILs* were planted in a greenhouse at the Jiangsu Academy of Agricultural Sciences (Nanjing, China). We used the small (S, 1–3 cm), medium (M, 4–6 cm), and large (L, 8–10 cm) panicle size for specimen collection. We also collected three biological repeats for each of these periods for each of the two *NILs* (*n* = 18). Spikelets were snap-frozen in liquid nitrogen following separation and stored at −80°C. The growth and development of these spikelets were highly consistent. We obtained grain length, width, and thickness measurements after harvesting and measured the thousand-grain weight after harvesting and drying at 37°C for 1 week. We observed epidermal cell morphology within the mature grain glumes with a scanning electron microscope (SEM; EVO-LS10, Carl Zeiss Microscopy GmbH, Oberkochen, Germany).

### RNA Extraction and Construction of a cDNA Library

Total RNA was extracted from the spikelet tissue using a TRIzol® RNA Purification Kit (Invitrogen, Carlsbad, CA, United States). We determined RNA purity, concentration, and integrity using a NanoDrop 2000 Spectrophotometer (ThermoFisher Scientific, Waltham, MA, United States), Qubit® 4.0 Spectrophotometer (Life Technologies, Carlsbad, CA, United States), and Agilent 2,100 Bioanalyzer® (Agilent Technologies, Palo Alto, CA, United States), respectively. The mRNA was then enriched using Oligo (dT) magnetic beads, and double-stranded cDNA (ds-cDNA) synthesis was performed. The ds-cDNAs were then purified using Agencourt AMPure XP beads (Beckmann Coulter, Pasadena, CA, United States), followed by end repair, polyadenylation, and sequencing connector. Finally, we used AMPure XP beads to screen for fragment size and polymerase chain reaction (PCR) amplification before constructing the sequencing library. The library was sequenced on an Illumina NovaSeq 6,000 sequencer in the PE150 mode (paired-end 150 bp).

### Long Non-coding RNA Prediction

We cleaned the raw sequences by removing adapter sequences, and low-quality reads with Q20 (a Phred quality score of 20 represents an error rate of 1 in 100) using the Fastp program (Chen et al., 2018), and eliminated any sequences ≤50 bp. The cleaned reads were then mapped to *Nipponbare* reference genome (https://rapdb.dna.affrc.go.jp, IRGSP-1.0 version) with the Hisat2 program (Kawahara et al., 2013; Pertea et al., 2016). We extracted unique reads (MAQ ≥20) for analysis using SAM tools v1.6 (Li et al., 2009). We updated the genome annotation with a reference annotation-based transcript (RABT) method, based on IRGSP-1.0 GTF annotation file (https://rapdb.dna.affrc.go.jp), using the StringTie program (Pertea et al., 2016). We extracted updated transcripts using the Gffreads program and performed a potential coding prediction using the CPC2 (Kang et al., 2017) and PLEK programs (Li et al., 2014). We considered transcripts with a length greater than 200 bp and consistently identified them as non-coding using both algorithms to be lncRNAs.

### Identification of Differentially Expressed Genes

We determined the expression abundance of each transcript based on the fragments per kilobase of transcript per million fragments mapped (FPKM) value and identified unexpressed or false positive transcripts using the largest FPKM value < 1 in multiple samples (Trapnell et al., 2010). We identified differentially expressed genes (DEGs) at different stages of panicle development using DESeq2 software (Love et al., 2014) with a Log2 (fold-change) ≥1 and q-value <0.05 required. We then visualized DEG commonalities between samples using a Venn plot.

### Fuzzy C-Means Clustering

Expressed genes from three development stages in *NILs* were grouped into different clusters using the Mfuzz package in R with fuzzy c-means (Kumar and Futschik, 2007). The different clusters represented the different expression patterns during rice panicle development. The gene expression values were standardized to have a mean value of zero and one standard deviation for each gene profile. The transformed expression values were then clustered using the fuzzy c-means clustering algorithm by the Mfuzz package.

### GO and KEGG Enrichment Analysis

The ClusterProfile package (Yu et al., 2012) was used for enrichment analysis and visualization using the Gene Ontology (GO) and Kyoto Encyclopedia of Genes and Genomes (KEGG) databases, with an FDR <0.05 required for significance. Heat maps of the gene expression profiles were generated using R packages.

### Validation by Quantitative Real-Time PCR

To validate the reliability of the RNA-Seq analysis, we performed quantitative real-time PCR (qRT-PCR) using ten randomly selected genes. The primers were designed using Primer Premier v.5.0 software (Premier Biosoft International, Palo Alto, CA, United States). We synthesized the first-strand cDNA using a TransScript One-Step gDNA Removal and cDNA Synthesis SuperMix Kit (TransGen Biotech, Beijing, China). We conducted the qRT-PCR on an ABI StepOnePlus Real-Time PCR System (Applied Biosystems, Foster City, CA, United States) using SYBR Premix Ex Taq (TaKaRa, Dalian, China). We used *Actin1* (Os03g0718100) as an internal standard to calculate the relative expression level and determined expression values based on the relative quantitative method (Livak and Schmittgen, 2001).

## Data Availability

The datasets presented in this study can be found in National Center for Biotechnology Information (NCBI) BioProject database under accession number PRJNA663252.

## Supplementary Material

Supplementary Figure S1 Quantitative analysis of 10 selected DEGs in RNA-Seq. Each bar represents SD; S, 526 young panicle 1–3 mm; M, young panicle 4–6 mm; L, young panicle 8–10 mm.
